# Angstrom-scale flatness using selective nanoscale etching

**DOI:** 10.3762/bjnano.8.217

**Published:** 2017-10-18

**Authors:** Takashi Yatsui, Hiroshi Saito, Katsuyuki Nobusada

**Affiliations:** 1 School of Engineering, University of Tokyo, Bunkyo-ku, Tokyo, 113-8656 Japan; 2 Department of Theoretical and Computational Molecular Science, Institute for Molecular Science, Myodaiji, Okazaki 444-8585 Japan

**Keywords:** Angstrom-scale flatness, optical near-field, wet etching

## Abstract

The realization of flat surfaces on the angstrom scale is required in advanced devices to avoid loss due to carrier (electron and/or photon) scattering. In this work, we have developed a new surface flattening method that involves near-field etching, where optical near-fields (ONFs) act to dissociate the molecules. ONFs selectively generated at the apex of protrusions on the surface selectively etch the protrusions. To confirm the selective etching of the nanoscale structure, we compared near-field etching using both gas molecules and ions in liquid phase. Using two-dimensional Fourier analysis, we found that near-field etching is an effective way to etch on the scale of less than 10 nm for both wet and dry etching techniques. In addition, near-field dry etching may be effective for the selective etching of nanoscale structures with large mean free path values.

## Introduction

The use of optical near-fields (ONFs) has contributed to the progress of nanoscale optical measurements [1], nanoscale fabrication [2], and photonic devices [3] below the diffraction limit of light. Recent ONF studies have exploited non-uniformity to realize new properties such as second harmonic generation [4], dipole-forbidden transitions [5], and indirect band transitions [6–8].

**Figure 1:**
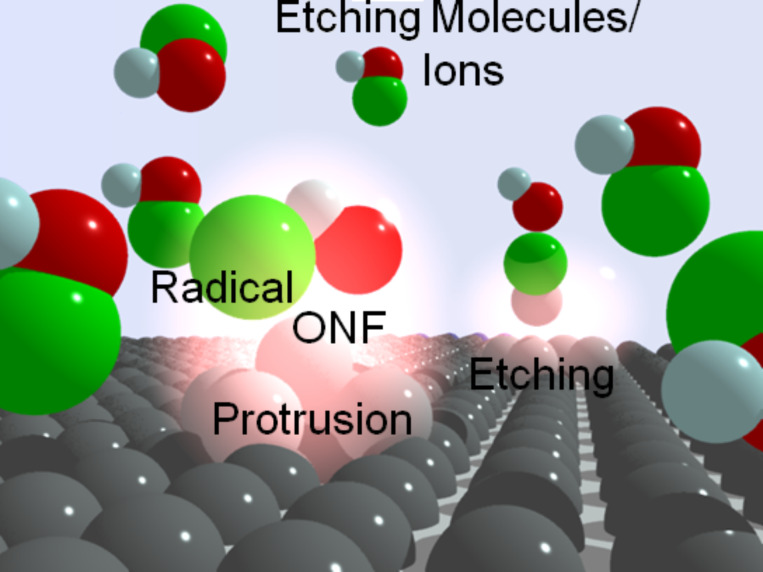
Schematic of the near-field etching process.

Because the ONF can achieve dipole-forbidden transitions, we can create selective chemical reactions that occur only where the ONF has been generated. Using this concept, we have developed a near-field etching technique. In this process, the molecules are dissociated by an ONF with a lower photon energy than that of the molecules (see Figure 1). When light irradiates the substrate, the ONF is generated only at the protrusions because of their non-uniformity. Subsequently, the molecules are dissociated by the ONF, and the dissociated radicals selectively etch the protrusions. Finally, the ONF disappears and the etching process stops automatically. Near-field etching is performed using Cl_2_ gas for glass, GaN [9], and plastic surfaces, and O_2_ gas for diamond and organic materials [10]. Atomically flat surfaces both on the flat regions and on three-dimensional structures has been achieved.

In this study, we compared near-field etching using a solution (wet etching) as well as dry etching. To evaluate the ONF effect with respect to wet or dry etching, we examined the etching characteristics using a two-dimensional Fourier analysis.

## Experimental

### Nanoscale etching

**Figure 2:**
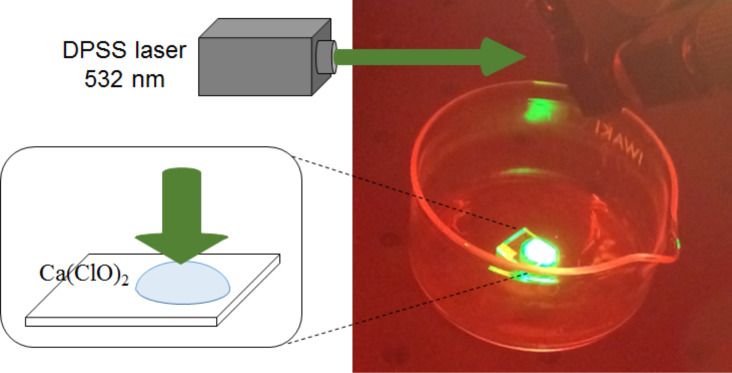
Experimental setup for the near-field wet etching technique.

For dry etching, we used Cl_2_ gas at a pressure of 200 Pa. 25 wt % calcium hypochlorous acid (Ca(ClO)_2_) was used as the source ion for wet etching. To dissociate the Cl_2_ or hypochlorous acid, we used a continuous-wave diode-pumped solid-state (DPSS) laser (λ = 532 nm; 2.33 eV; excitation power: 119.4 W/cm^2^). Thus, the incident photon energy was lower than the dissociation energy of Cl_2_ (3.10 eV) [11] and the hypochlorous acid (3.35 eV) [12]; therefore, the Cl_2_ or hypochlorous acid dissociated on the protrusions only. In the solution, a light source with a photon energy of 4.66 eV (higher than the dissociation energy) dissociated the hypochlorous acid and consequently produced Cl radicals [13]. This process is expected to be similar to the etching of glass when Cl_2_ gas is used. The laser light was incident from the top of the substrate through a chamber with 200 Pa Cl_2_ for dry etching or a drop of 10 μL Ca(ClO)_2_ (25 wt %) for wet etching (see Figure 2). The absorption edge of fused silica is approximately 7.9 eV (157 nm) [14]; thus, we could exclude the effect of carrier generation in the fused silica. To evaluate the changes in the surface profiles, we used an atomic force microscope (AFM) with a “Sampling Intelligent Scan” mode (Hitachi-Hitech-Science Corp.). The scanning area of the AFM was 10 × 10 μm and 256 × 256 pixels.

## Results and Discussion

**Figure 3:**
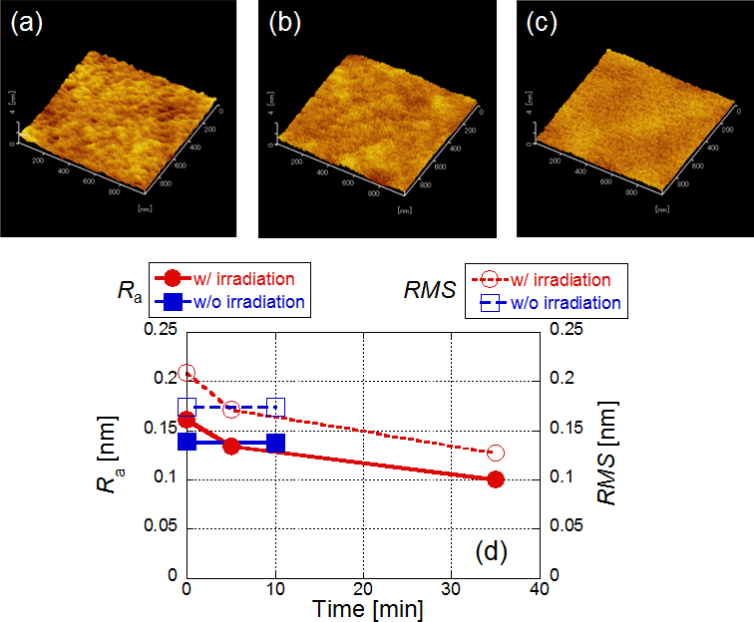
Time dependence of surface roughness using a solution (wet etchng). AFM images taken (a) before etching, (b) after 5 min, and (c) after 35 min. (d) Time dependence of the value of *R*_a_. Red solid circles: with irradiation, blue solid squares: without irradiation. Time dependence of the value of RMS (right hand side). Red open circles: with irradiation, blue open squares: without irradiation.

Figure 3a–c shows the respective AFM images obtained 0 min, 5 min, and 35 min after wet etching. In the AFM images, the surface roughness (*R*_a_) was found to be 0.161, 0.134, and 0.100 nm, respectively (solid circles in Figure 3d). In addition, we checked the *R*_a_ where light was not irradiated with the Ca(ClO)_2_ solution and found that its value was unchanged (0.139 nm for before and 0.138 nm for after etching; solid squares in Figure 3d). These results indicate that near-field wet etching decreased the surface roughness. We also plotted the root mean square (RMS) roughness values in Figure 3d. Although the value is not the same, they have a similar time dependence as *R*_a_.

**Figure 4:**
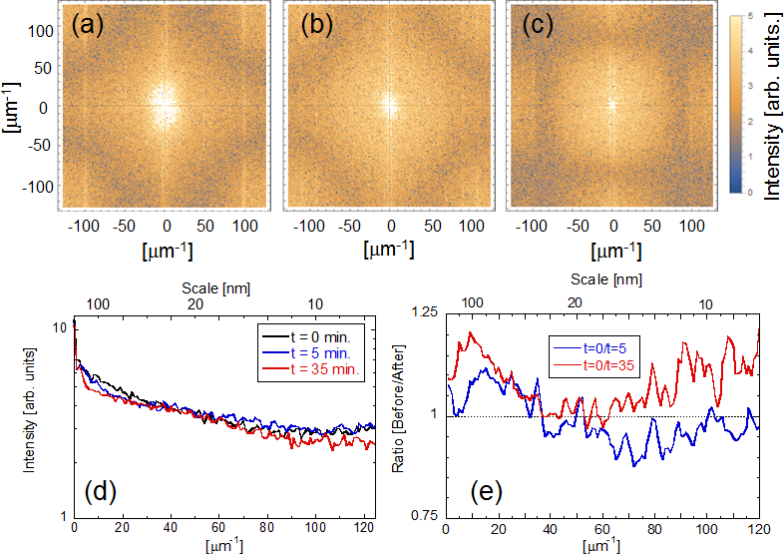
Time dependence of Fourier power spectra using a solution. Fourier power spectra taken (a) before etching, (b) after 5 min, and (c) after 35 min. (d) Averaged cross-sectional profiles. (e) Ratio of the power spectra before etching to the power spectra after etching. Blue solid line: before (*t* = 0 min)/after (*t* = 5 min), red solid line: before (*t* = 0 min)/after (*t* = 35 min).

To evaluate the etching properties, we calculated two-dimensional Fourier power spectra. Figure 4a–c shows the respective Fourier power spectra calculated from Figure 3a–c, respectively. To find the differences, the averaged cross-sectional profiles are plotted in Figure 4d. In addition, Figure 4e shows the ratio of the power spectra before etching to the power spectra after and before etching. A decrease in this ratio implies the etching was successful. These results show that at the beginning of the near-field wet etching, larger scale areas (more than 30 nm) were etched, and after that, smaller scale areas (less than 15 nm) were also etched.

**Figure 5:**
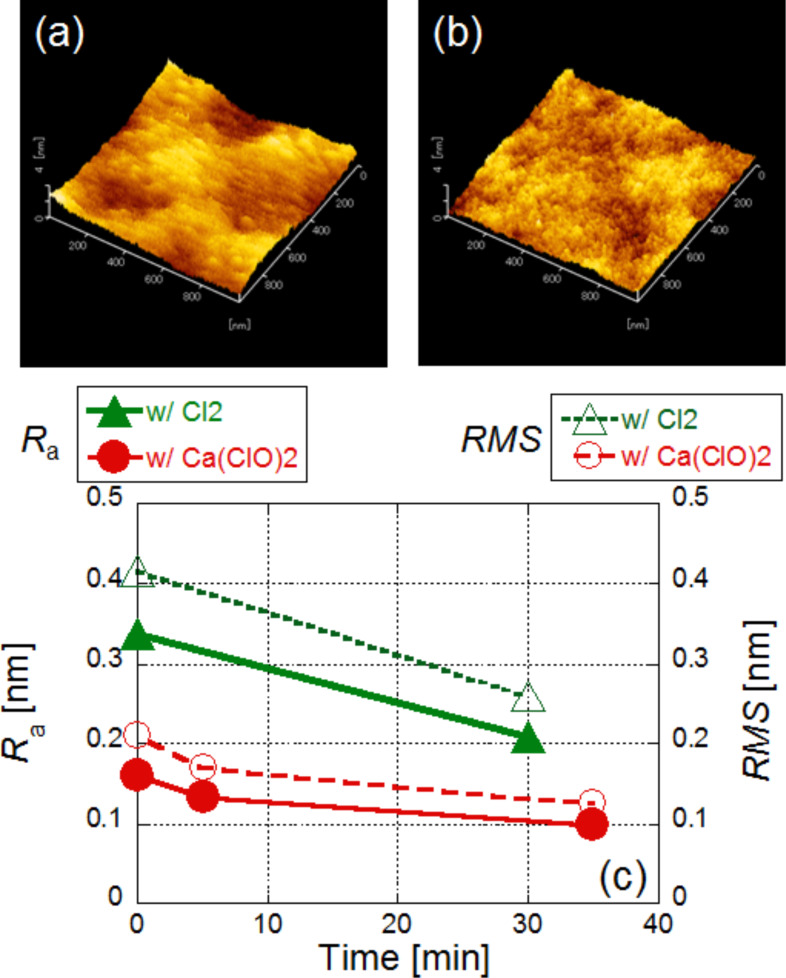
Time dependence of surface roughness. AFM images taken (a) before etching and (b) after 30 min. (c) Time dependence of the value of *R*_a_. Red solid circles: with Ca(ClO)_2_, green solid triangles: with Cl_2_. Time dependence of the value of RMS. Red open circles: with Ca(ClO)_2_, green open triangles: with Cl_2_.

**Figure 6:**
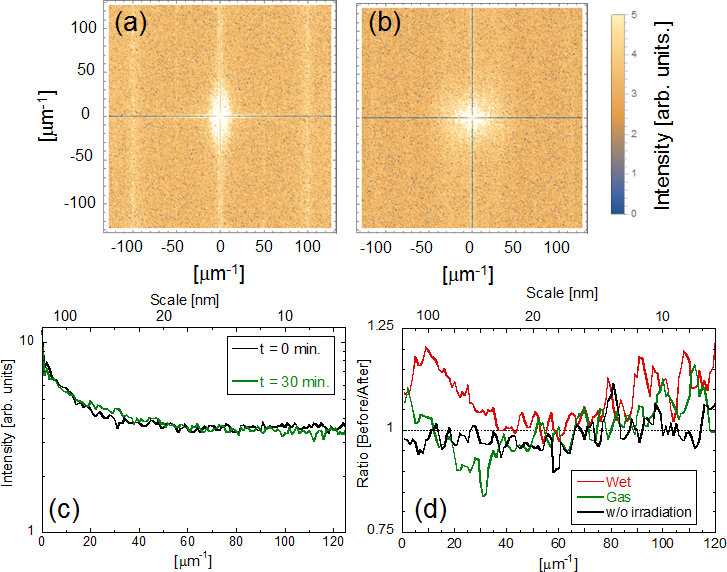
Time dependence of Fourier power spectra using a gas. Fourier power spectra taken (a) before etching and (b) after 30 min. (c) Averaged cross-sectional profiles. (d) Ratio of the power spectra before to the power spectra after etching. Red solid line: before (*t* = 0 min)/after (*t* = 35 min) etching using a solution, black solid line: before (*t* = 0 min)/after (*t* = 10 min) etching using a solution without irradiation, green solid line: before (*t* = 0 min)/after (*t* = 30 min) etching using a gas.

We compared the etching properties obtained from these results with those from a near-field dry etching technique. For this purpose, we obtained AFM images before and after near-field etching using Cl_2_ gas. Figure 5a,b shows images 0 min and 30 min after etching. The AFM images show that the *R*_a_ was reduced from 0.338 nm to 0.208 nm (solid triangles in Figure 5c). We plotted RMS values in Figure 5c and found a similar time dependence as with *R*_a_. We obtained the two-dimensional Fourier power spectra from the AFM images. Figure 6a,b shows the respective power spectra before and after near-field etching using Cl_2_. To find the differences, the averaged cross-sectional profiles are plotted in Figure 6c. In addition, Figure 6d shows the ratio of the power spectra before etching to the power spectra after etching (green solid line). In this figure, we also compare the same ratio for wet etching (red solid line) and without irradiation (solid black line). These results show that although the ratio did not change in the absence of irradiation, smaller scale areas less than 10 nm were also etched in the near-field dry etching process.

To understand the differences of the etching properties, we estimated the mean free paths (MFPs) of Cl_2_ and ClO^−^. The MFP of Cl_2_ in the gas phase is on the order of 10 μm (≈*RT*/√2*N*_A_π*d*^2^*P* [15], where *R* = 8.31 J K^−1^mol^−1^, *T* = 300 K, *N*_A_ = 6.02 × 10^23^ mol^−1^, *d* = 200 × 10^−12^ m (the diameter of a Cl_2_ molecule) and *P* = 200 Pa). In contrast, the MFP of ions in solution is on the order of 10 pm [16], which is 10^−7^ times smaller than that of Cl_2_ gas. Such a low MFP value for ions in solution resulted in etching near the protrusions where the ions or molecules are dissociated. However, in the case of near-field dry etching, because of a greater MFP value of the gas phase that exceeds the scanning area, the dissociated atoms can react only when they are located at the protrusions where the ONF generated.

## Conclusion

Using two-dimensional Fourier analysis, we found that near-field etching is effective for etching on smaller scales less than 10 nm for both wet and dry etching. In addition, by comparing wet and dry etching, near-field dry etching was shown to be effective for the selective etching of nanoscale structures because of the large value of the mean free path of the etching molecules. Hence, further control of the selective etching at smaller scales should be achievable by controlling mean free path (i.e. the partial pressure of the gas).
